# Prenatal Betamethasone Exposure and its Impact on Pediatric Type 1 Diabetes Mellitus: A Preliminary Study in a Spanish Cohort

**DOI:** 10.1155/2022/6598600

**Published:** 2022-03-10

**Authors:** David Perna-Barrull, Marta Murillo, Nati Real, Laia Gomez-Muñoz, Silvia Rodriguez-Fernandez, Joan Bel, Manel Puig-Domingo, Marta Vives-Pi

**Affiliations:** ^1^Immunology Service Germans Trias i Pujol Research Institute and University Hospital, Autonomous University of Barcelona, 08916 Badalona, Spain; ^2^Pediatrics Service Germans Trias i Pujol Research Institute and University Hospital, Autonomous University of Barcelona, 08916 Badalona, Spain; ^3^Endocrinology Service Germans Trias i Pujol Research Institute and University Hospital, Autonomous University of Barcelona, 08916 Badalona, Spain

## Abstract

**Background:**

Betamethasone, a glucocorticoid used to induce lung maturation when there is a risk of preterm delivery, can affect the immune system maturation and type 1 diabetes (T1D) incidence in the progeny. It has been described that prenatal betamethasone protects offspring from experimental T1D development. The main aim of this study was to evaluate the possible association between betamethasone prenatal exposure and T1D in humans. *Research Design and Methods*. A retrospective case-control study with a total of 945 children, including 471 patients with T1D and 474 healthy siblings, was performed. Participants were volunteers from the Germans Trias i Pujol Hospital and DiabetesCero Foundation. Parents of children enrolled in the study completed a questionnaire that included questions about weeks of gestation, preterm delivery risk, weight at birth, and prenatal betamethasone exposure of their children. Multiple logistic regression was used to detect the association between betamethasone exposure and T1D.

**Results:**

We compared T1D prevalence between subjects prenatally exposed or unexposed to betamethasone. The percent of children with T1D in the exposed group was 37.5% (21 of 56), and in the unexposed group was 49.52% (410 of 828) (*p* = 0.139). The percentage of betamethasone-treated subjects with T1D in the preterm group (18.05%, 13 of 72) was significantly higher than that found in the control group (12.5%, 9 of 72) (*p* = 0.003). The odds ratio for T1D associated with betamethasone in the univariate logistic regression was 0.59 (95% confidence interval, 0.33; 1.03 [*p* = 0.062]) and in the multivariate logistic regression was 0.83 (95% confidence interval, 0.45; 1.52 [*p* = 0.389]).

**Conclusions:**

The results demonstrate that the prenatal exposure to betamethasone does not increase T1D susceptibility, and may even be associated with a trend towards decreased risk of developing the disease. These preliminary findings require further prospective studies with clinical data to confirm betamethasone exposure effect on T1D risk.

## 1. Introduction

Type 1 diabetes (T1D) is amongst the most common endocrine disorder in children and adolescents. Over the last decades, the incidence of T1D during childhood is increasing with an average of 3–4% per year [1]. T1D is caused by the autoimmune destruction of pancreatic *β*-cells, and despite genetic risk factors have been identified, the influence of environmental factors is of foremost importance and has been exhaustively studied in large patient cohorts such as TEDDY study (The Environmental Determinants of Diabetes in the Young) [2], or DIPP (Type 1 Diabetes Prediction and Prevention) study [3]. Among them, environment-related prenatal changes might have an influence on the development of T1D. In fact, the in utero environment is critical for disease development, as suggested by the fact that dizygotic twins display an increased concordance of T1D when compared to nontwin siblings [4]. Environmental factors in utero such as iron overload or low zinc concentration in drinking water [5, 6] correlate with an increased risk to develop autoimmune diabetes. Moreover, the gestational age has impact on the T1D risk. In general terms, preterm newborns showed an increased risk to develop the disease [7, 8] but some controversy exists regarding very preterm birth (birth before 32 weeks of gestation) that seems to protect against T1D [9].

Preterm birth, defined as birth occurring at less than 37 weeks of gestation, is a serious and common pregnancy complication reaching 5–11% of live births in developed countries [10, 11]. Administration of antenatal corticosteroids is recommended as the standard care for the management of women at risk of imminent preterm delivery [12–14]. However, the benefits and harms of such a strategy are still under discussion, especially when administered after week 34 of gestation or before cesarean delivery [15, 16].

Specifically, synthetic glucocorticoids, most often betamethasone, are routinely administered to women at risk of preterm delivery between 24 and 34 weeks of gestation to accelerate lung maturation and reduce the severity of respiratory distress syndrome, therefore improving the survival rates of premature infants. Recent studies in experimental models of T1D show that prenatal betamethasone affects the two main players in this disease, the immune system and the pancreatic *β*-cells, and that corticosteroid-related changes may have long-term consequences in the offspring [17–19]. Since both the immune system and the pancreatic islets are still developing until birth, glucocorticoid exposure [20] may affect islet function [21] and T1D susceptibility. Data from both experimental and clinical studies suggests a link between prenatal exposure to glucocorticoids and alterations in immune traits in the offspring [22]. In summary, antenatal glucocorticoids exert an anti-inflammatory effect, contributing to a switch towards a Th2 response.

The main aim of the current study was to evaluate the possible association between prenatal betamethasone exposure and T1D development in humans, as a previous step for future studies on human cohorts that will help to elucidate the role of this drug in the susceptibility to T1D.

## 2. Materials and Methods

### 2.1. Design

This is a retrospective case-control study conducted between 2018 and 2021 in Spain. A questionnaire was carried out to evaluate the possible association between prenatal betamethasone exposure and the T1D development. A total of 945 children participated in the study. The primary population explored was a group of 471 children being diagnosed with T1D, which was compared with a control group constituted by their sisters and brothers (*n* = 474). Within these two groups, betamethasone treatment (two doses of 12 mg, 24 h apart) [13] at the time of late gestation was assessed in order to elucidate whether betamethasone could have a protective effect against T1D development in those children prenatally exposed to the drug. Parents of children enrolled in the study were requested to complete a questionnaire that included questions about gender, diagnosis of T1D, age at diagnosis, weeks of gestation, weight at birth, and prenatal betamethasone exposure of their children (Table 1). The questionnaire was conducted in two settings: in the Pediatrics Service of the Germans Trias i Pujol Hospital (Badalona, Spain) and in DiabetesCero Foundation (Spain), a non-profit organization made up of parents of children with T1D. Questionnaire forms were distributed by email or in person. An electronic database was set-up to record entries in submitted questionnaire forms. The questionnaire was approved by the Ethics Committee for Research at the Germans Trias i Pujol Hospital. All participants were informed of the nature and purpose of the questionnaire at the outset, and informed consent was obtained from legal representatives of all participants.

### 2.2. Sample Size and Statistical Methods

The sample size for the study was calculated using the Cochran's formula. Since there are no previous data on similar studies, we calculated the number of subjects per group with a statistical value (*P*-value) of 0.05 and a potency value of 0.8. To that end, the estimated proportions for the control group were obtained from both bibliographic review of Spanish preterm birth rate studies (that account for 8.7% of total births) [23] and from the percentage of antenatal corticosteroids administered in those cases (62% of women at risk of preterm delivery are treated with betamethasone) [24]. This resulted in an estimated proportion for the control group of 5.22% subjects exposed to betamethasone. The estimated proportions for the T1D group (*P*_1_) were calculated using the formula *P*_1_ = OR∗*P*_2_/(1-*P*_2_) + (OR∗*P*_2_). In order to obtain the odds ratio (OR), previous results on the offspring of nonobese diabetic (NOD) mice after betamethasone exposure were used (OR = 0.287) [17]. The *P*_2_ was obtained from the already calculated proportion of betamethasone-exposed individuals in the control group (*P*_2_ = 0.0522). The formula offered an estimated proportion for the T1D group of 1.56% subjects exposed to betamethasone. Cochran's formula comprising the calculated proportions (*P*_1_ and *P*_2_) resulted in a sample size of 378 subjects per group.

Statistical analysis was performed using Prism 9.0 software (GraphPad Software INC., San Diego, CA, USA) and statistical software R (Vienna, Austria). Descriptive statistics were applied to the collected data. Continuous variables (weeks of gestation and birth weight) were analyzed using a *T*-test or an analysis of variance (ANOVA) to compare between variables. Categorical variables (T1D, exposure to betamethasone and gender) were analyzed using a chi-squared test to compare between variables. For correlations, a nonparametric Spearman's test was performed. To compare T1D prevalence between betamethasone exposed and unexposed subjects a Logrank Mantel-Cox test was performed.

Multiple logistic regression was used to detect the association between betamethasone exposure and T1D after adjusting for gender, weeks of gestation, and birth weight. Bioinformatic analysis was carried out by the Statistics and Bioinformatics Unit (UEB) from Vall d'Hebron Research Institute (VHIR, Barcelona, Spain). Subjects with missing values for one or more variables were not added in the statistical analysis. Values with a *p* < 0.05 were considered statistically significant.

## 3. Results

Data from 945 children were collected (Table 1) and classified into two groups: patients with T1D diagnosed during childhood or puberty (T1D group) and their siblings (control group). Males were slightly predominant over females in both groups (52.3% and 47.7% vs. 52.2 and 47.8%, respectively). The gender was missing in 14.6% of total individuals. The age at T1D diagnosis was 7.42 ± 4.94 years (mean ± SD). Children with T1D were born after a significantly and slightly longer gestation period than control group (39.02 ± 1.93 weeks vs. 38.56 ± 2.22 weeks; *p* = 0.0011), but no differences were observed in the birth weight between groups. Only 5.93% (56 of 945) of the subjects in the study were prenatally exposed to betamethasone, and a significantly lower number of children who received prenatal betamethasone were in the T1D group (4.73%, 21 of 471), when compared to the control group (7.77%, 35 of 474) (*p* = 0.0166). Similarly, a signifanctly lower number of preterm babies was found in the T1D group when compared to controls (8.70% vs. 13.07%; *p* = 0.0145).

**Figure 1:**
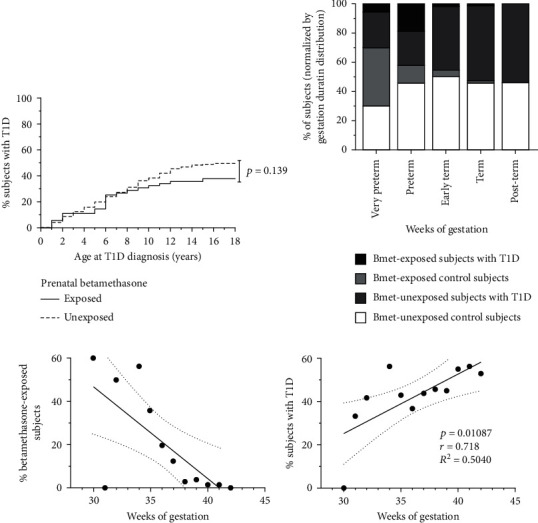
Prenatal betamethasone exposure tends to reduce the percentage of subjects with type 1 diabetes. (a) Percentage of subjects with type 1 diabetes (T1D) in children prenatally exposed to betamethasone (continuous line, *n* = 56) and in unexposed children (dotted line, *n* = 828). Logrank Mantel-Cox test was used for statistical analysis. (b) Percentage of children in each gestational period: very preterm (28–32 week gestational age (GA), *n* = 20); preterm (33–36 week GA, *n* = 72); early term (37–38 week GA, *n* = 248); term (week 39–41 GA, *n* = 470); postterm (≥42 week GA, *n* = 50). White bars represent the percentage of the betamethasone-unexposed (Bmet-unexposed) control subjects; light grey bars represent the percentage of betamethasone-exposed (Bmet-exposed) control subjects; dark grey bars represent the percentage of Bmet-unexposed subjects with T1D; and black bars represent the percentage of Bmet-exposed subjects with T1D. All percentages were calculated with respect to the total number of subjects in each gestational period. (c) Percentages of children prenatally exposed to betamethasone in relation to weeks of gestation. (d) Correlation between the percentages of children with T1D and weeks of gestation (Spearman's correlation analysis).

First, we compared the prevalence of T1D between both groups.  Figure 1(a) shows that the percentage of subjects with T1D of the exposed group was 37.5% (21 of 56), and the percentage of subjects with T1D of the unexposed group was 49.52% (410 of 828). Despite nonsignificant (*p* = 0.139), this result seems to suggest a biological trend.

The percentage of subjects exposed to betamethasone with or without T1D depending on gestational age is shown in Figure 1(b). Regarding prematurity (less than 37 weeks of gestational age), we observed a significantly higher percentage of preterm babies in the control group (13.07%, 60 of 474) in comparison to the T1D group (8.70%, 41 of 471) (*p* = 0.0145). The percentage of subjects with T1D and controls exposed to betamethasone was represented in each subgroup of gestation duration. As expected, in the control group the highest percentage of betamethasone exposure was found in the very preterm condition. Surprisingly, in the T1D group, we observed a higher percentage of betamethasone exposure in the preterm group (18.05%, 13 of 72) when compared to the very preterm group (5%, 1 of 20) whereas in the control group this value decreased with the duration of gestation. Moreover, this high percentage of betamethasone-treated T1D subjects (18.05%, 13 of 72) in the preterm group is significantly higher than that found in the control group (12.5%, 9 of 72) (*p* = 0.003). The percentage of betamethasone exposure in deliveries from 37 weeks of gestation showed a tendency to decrease in the T1D group when compared to controls: early term (1.21 vs. 4.44%, 3 and 11 of 248, respectively) and term (0.85 vs. 1.28%, 4 and 6 of 470, respectively) (Figure 1(b)). As expected, a high percentage of preterm newborns received prenatal betamethasone (Figure 1(c)). A direct correlation was found between weeks of gestation and T1D in the offspring (Figure 1(d)).

As detailed in Supplementary Figure 1A, the predominant age at clinical onset was between 1 and 12 years. With regard to children in the T1D group that were exposed to betamethasone *in utero*, the majority of them were diagnosed at an early age (from 0 to 7 years) (5.88%, 15 of 255), whereas a minority was diagnosed with the disease at a later age (from 8 to 17 years) (2.97%, 6 of 202) (Supplementary Figure 1B).

Then, we analyzed perinatal data depending on the exposure to betamethasone. As expected, betamethasone-exposed children displayed a lower birth weight and fewer weeks of gestation than unexposed children (*p* = 0.00001) (Table 2). Most children (85.25%, 763 of 895) had appropriate weight for gestational age, according to the Spanish birthweight charts [25]. Interestingly, we found that prenatal exposure to betamethasone was more frequent in males than in females (*p* = 0.0257) (Table 2).

**Figure 2:**
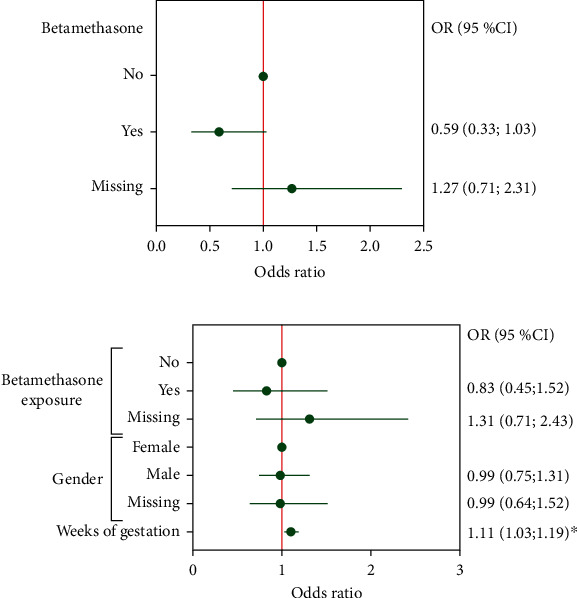
Prenatal betamethasone administration and type 1 diabetes risk. (a) Forest plot of the univariate logistic regression odds ratios (OR) and 95% confidence interval (95% CI) for the risk of type 1 diabetes (T1D) according to prenatal betamethasone exposure. (b) Forest plot of the multivariate logistic regression OR and 95% CI for the risk of T1D according to prenatal betamethasone exposure, gender, or week of gestation. T1D OR was adjusted for birth weight, gender, and weeks of gestation. ∗*p* < 0.01, multiple logistic regression.

Finally, we analyzed the data using a logistic regression model. First, we used a univariate approach between betamethasone and T1D development. The odds ratio (OR) for T1D associated with betamethasone was 0.59 (95% confidence interval [95% CI], 0.33; 1.03 [*p* = 0.062]). Although nonsignificant, a trend towards a protective effect of betamethasone against T1D development was observed (*p* = 0.062, Figure 2(a)). Then, we adjusted the betamethasone effects in a multivariate analysis. The variables weeks of gestation and birth weight were considered as confounding factors. The effect of betamethasone on T1D displayed the same trend, although less evidently (OR 0.83 [95% CI, 0.45; 1.52] [*p* = 0.389]). By contrast, the effect of weeks of gestation on T1D showed a statistically significant OR of 1.11 (95% CI, 1.03; 1.19 [*p* = 0.0052]) (Figure 2(b)).

## 4. Discussion

In this study, we address for the first time the relationship between prenatal administration of betamethasone and T1D development in the offspring. Previous results of our group demonstrated a protective role of prenatal betamethasone exposure against T1D in NOD mice [17]. The chosen strategy to evaluate the effect of this drug in humans was a questionnaire for parents of children with T1D, whereas the control group consisted of healthy siblings or twins of a subject with the disease. Betamethasone exposure did not increase the susceptibility to T1D and was even associated with a nonsignificant decreased risk of T1D. This association seems to be higher in children diagnosed with T1D under 8 years of age. Because very young children usually have an aggressive form of T1D [26], it is reasonable to assume that the severity of autoimmunity would be much more difficult to halt or that the genetic component is stronger in these children [27]. We have to take into account that the control group was composed of siblings of patients with T1D, in which the risk to develop T1D is more than 10 times higher than in the general population [28].

The effects of glucocorticoids, administered during pregnancy, on childhood diabetes were previously reported in a Danish cohort study [29]. This study showed that prenatal exposure to corticosteroids tends to increase T1D incidence in the offspring. Nevertheless, there are substantial differences between this study and the present work. Whereas we started with a cohort of T1D children and siblings (*n* = 945), Greene et al. started with approximately 500,000 children born in Denmark in a specific period (between 1 January 1997 and 31 December 2004). So, our cohort is smaller than the Danish one but includes 50% of T1D patients, in comparison to less than 1% of Danish children with T1D. Importantly, in the Danish cohort the authors took into account not only prenatal betamethasone administration but also glucocorticoid exposure (topical, inhaled, and systemic corticosteroids) throughout the pregnancy. Only 4.5% of the prenatally glucocorticoid-exposed children received one or more systemic glucocorticoids (betamethasone and other synthetic glucocorticoids). In this sense, we believe that our study was focused on the effect of a single course of prenatal systemic betamethasone exposure in the third trimester. The duration of the exposure before giving birth could prove crucial because betamethasone and its derivatives have a half-life of approximately 3 days in the human body [30]. We hypothesize that repeated doses of this drug in a short span of time would increase exponentially the glucocorticoid concentration, thus inducing side effects such as hyperglycemia and insulin resistance [31], among others [22, 32]. On the other hand, perinatal betamethasone effects related to T1D could differ depending on the gestational week of administration. For these reasons, we consider that our results point to a different direction. In fact, antenatal glucocorticoids have an important impact on immune system ontogeny [22] and modify the T cell receptor repertoire, as we demonstrated in NOD mice [17]. Our recent results also showed betamethasone effects on *β*-cell growth, metabolism, and immunogenicity [19]. Additional effects of glucocorticoids are epigenetic modifications [33] which could affect both the developing immune system and the *β*-cells. These effects have positive consequences on self-tolerance in NOD mice, in this regard, betamethasone might even be a factor that could help to tip the balance towards *β*-cell tolerance, thus contributing to prevent autoimmunity in subjects at risk of developing T1D.

Interestingly, and independently of the effect of betamethasone, the T1D group of this study shows a longer pregnancy duration than the control group. Other studies do not report any effect [34] or point to the opposite: preterm and early term delivery correlate to an increased risk of T1D, whereas post-term delivery is associated with a reduction in T1D risk [7, 35, 36]. These controversial differences can be due to geographical and demographic factors, but also to the analysis performed in our study. Here, weeks of gestation of the control group and children with T1D were compared, whereas other reported studies analyzed the incidence of T1D in the 4–5 different gestational periods. The reported difference is only of 3 days, and despite it being statistically significant, it could be a coincidental finding.

We are well aware of the limitations of our study, including the use of a questionnaire instead of clinical data registry. However, due to technical and ethical issues, it was impossible to link both the medical registries of the mothers and their children in our study population. Another limitation was the recall bias in the questionnaire, particularly for the betamethasone exposure, which is our main focus. We have determined that 5% of mothers do not remember whether they received betamethasone. To avoid it, we have defined a specific target population, and the questionnaire has been designed as short, precise, and accessible as possible. Nonetheless, this “missing” group gives similar perinatal data than the unexposed betamethasone group, so it is unlikely that group participants received betamethasone at late pregnancy. In addition, participants were recruited from two sources, but the incidence of T1D in the two geographical areas where the questionnaire was performed is similar (questionnaires obtained from Germans Trias i Pujol Hospital belong to Catalonia region and from DiabetesCero foundation belong to all Spanish regions) [37, 38]. The fact that the control group is composed of siblings of patients with T1D is a particular feature of the study, aiming at minimizing the variations due to environmental factors.

However, additional results from the present study fit well with previous reports, thus validating the study group. First, a significantly higher number of preterm newborn males was observed as compared to their female counterparts. This finding has already been reported in previous studies [39, 40]. Second, as expected, betamethasone-exposed newborns had lower birth weight and fewer gestational weeks than unexposed newborns.

## 5. Conclusion

Our study suggest that the prenatal exposure to betamethasone does not increase the susceptibility to T1D, and may even be associated with a trend towards decreased risk of developing this disease. However, these preliminary findings require further prospective studies with clinical data registries involving a larger sample size to draw definitive conclusions regarding the effects of prenatal betamethasone exposure on the risk of developing T1D in later life.

## Figures and Tables

**Table 1:** Clinical characteristics of the subjects in the study and control groups.

Characteristics	T1D group	Control group	*p* value
Number, *n* (%)	471 (49.8)	474 (50.2)	
Females, *n* (%)	192 (47.8)^†^	193 (47.7)^†^	0.977^‡^
Males, *n* (%)	210 (52.2)^†^	212 (52.3)^†^	0.977^‡^
Age at diagnosis (years, mean ± SD)	7.42 ± 4.94	NA	
Gestation weeks (mean ± SD)	39.02 ± 1.92	38.56 ± 2.22	0.0011^§^
Birth weight (kg, mean ± SD)	3.24 ± 0.52	3.21 ± 0.61	0.389^§^
Betamethasone, *n* (%)	21 (4.73)	35 (7.77)	0.0166^‡^
Preterm birth (≤36 weeks of gestation), *n* (%)	41 (8.70)	60 (13.07)	0.0145^‡^

^†^Missing gender accounts for 14.65% (69 patients) in subjects with T1D and 14.56% (69 subjects/participants) in control subjects. Subjects with missing gender have not been considered for gender ratio determination. ^‡^Chi-squared test; ^§^*T*-test. NA: not applicable; T1D: type 1 diabetes; SD: standard deviation.

**Table 2:** Variable distribution depending on betamethasone treatment.

Variable		Betamethasone			Total	*p*-value
		No	Yes	Missing		
Birth at weight^‡^ (kg, mean ± SD) (P25; P75)		*n* = 822 3.26 ± 0.53 (2.94; 3.60)	*n* = 56 2.63 ± 0.80 (2.02; 3.19)	*n* = 47 3.28 ± 0.53 (2.85; 3.66)	*n* = 925 3.23 ± 0.57 (2.90; 3.60)	0.00001^¶^
Duration of gestation^§^ (weeks, mean ± SD) (P25; P75)		*n* = 805 38.97 ± 1.93 (38.00;40.00)	*n* = 56 35.7 ± 2.87 (34.00;38.00)	*n* = 45 38.64 ± 1.97 (37.00;40.00)	*n* = 906 38.79 ± 2.09 (38.00;40.00)	0.00001^¶^
Sex	Female, *n* (%)	340 (40.4%)	19 (33.9%)	26 (54.2%)	385 (40.8%)	0.0257^||^
	Male, *n* (%)	374 (44.5%)	26 (46.4%)	22 (45.8%)	422 (45.3%)	
	Missing, *n* (%)	127 (15.1%)	11 (19.6%)	0 (0%)	138 (13.9%)	

^‡^ Birth weight was missing in 20 subjects (2.1%). ^§^ Duration of gestation was missing in 39 subjects (4.1%) ^¶^: ANOVA (analysis of variance). ^||^: chi-squared test.

## Data Availability

The data used to support the findings of this study are available from the corresponding author upon request.

## Supplementary Materials

Supplementary Materials Supplementary Figure 1: Prenatal administration of betamethasone and type 1 diabetes according to age. (A) Histogram of the number of subjects diagnosed with type 1 diabetes (T1D) in different age groups. White color corresponds to subjects unexposed to prenatal betamethasone, black color corresponds to subjects exposed to prenatal betamethasone, whereas grey color represents subjects whose status is missing (regarding the prenatal exposure to betamethasone). (B) Histogram of the percentage of betamethasone-exposed subjects in the T1D group depending on the age at disease diagnosis. Black bar corresponds to early onset of T1D (from 0 to 7 years, *n* = 255), while white bar corresponds to late onset of T1D (from 8 to 17 years, *n* = 202). Chi-squared test was used for statistical analysis.
