# Supplementary material for: Prenatal Betamethasone Exposure and its Impact on Pediatric Type 1 Diabetes Mellitus: A Preliminary Study in a Spanish Cohort
Source: J Diabetes Res. 2022 Mar 10;2022:6598600. doi: 10.1155/2022/6598600 (PMC8930272; doi:10.1155/2022/6598600)
Supplement: Supplementary Materials — Supplementary Figure 1: Prenatal administration of betamethasone and type 1 diabetes according to age. (A) Histogram of the number of subjects diagnosed with type 1 diabetes (T1D) in different age groups. White color corresponds to subjects unexposed to prenatal betamethasone, black color corresponds to subjects exposed to prenatal betamethasone, whereas grey color represents subjects whose status is missing (regarding the prenatal exposure to betamethasone). (B) Histogram of the percentage of betamethasone-exposed subjects in the T1D group depending on the age at disease diagnosis. Black bar corresponds to early onset of T1D (from 0 to 7 years, n = 255), while white bar corresponds to late onset of T1D (from 8 to 17 years, n = 202). Chi-squared test was used for statistical analysis. [file 6598600.f1.docx]

Prenatal betamethasone exposure and its impact on pediatric type 1 diabetes mellitus: A preliminary study in a Spanish cohort

**Supplementary Material**

David Perna-Barrull^1^, Marta Murillo^2^, Nati Real^2^, Laia Gomez-Muñoz^1^, Silvia Rodriguez-Fernandez^1^, Joan Bel^2^, Manel Puig-Domingo^3^, Marta Vives-Pi^*1^

^1^ Immunology Service Germans Trias i Pujol Research Institute and University Hospital, Autonomous University of Barcelona, 08916 Badalona, Spain.

^2^ Pediatrics Service Germans Trias i Pujol Research Institute and University Hospital, Autonomous University of Barcelona, 08916 Badalona, Spain.

^3^ Endocrinology Service Germans Trias i Pujol Research Institute and University Hospital, Autonomous University of Barcelona, 08916 Badalona, Spain

**Author for correspondence:** Marta Vives-Pi. Immunology Section, Germans Trias i Pujol Research Institute. Carretera Canyet s/n. 08916 Badalona, Spain

Phone:+34935543050, e-mail: [mvives@igtp.cat](mailto:mvives@igtp.cat)

***
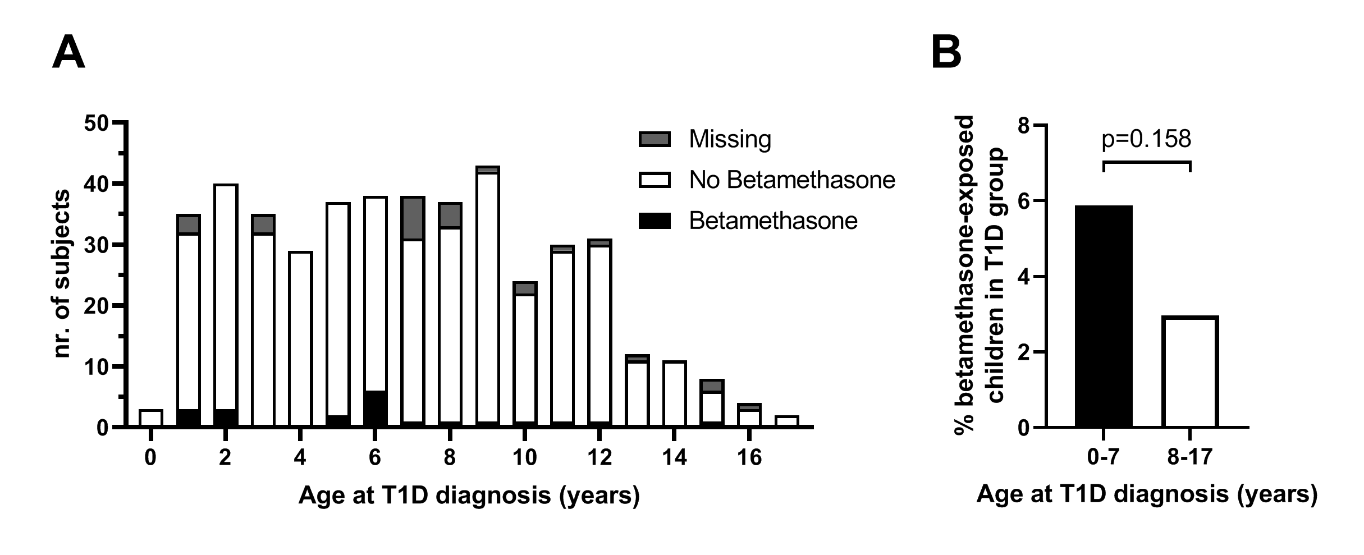
Supplementary Figure 1. Prenatal administration of betamethasone and type 1 diabetes according to age. A)*** *Histogram of the number of subjects diagnosed with type 1 diabetes (T1D) in different age groups. White color corresponds to subjects unexposed to prenatal betamethasone, black color corresponds to subjects exposed to prenatal betamethasone, whereas grey color represents subjects whose status is missing (regarding the prenatal exposure to betamethasone).* ***B)*** *Histogram of the percentage of betamethasone-exposed subjects in the T1D group depending on the age at disease diagnosis. Black bar corresponds to early onset of T1D (from 0 to 7 years, n=255), while white bar corresponds to late onset of T1D (from 8 to 17 years, n=202). Chi-squared test was used for statistical analysis.*
